# Neuroimaging appearance of hypothalamic hamartomas in monozygotic twins with Pallister-Hall syndrome: case report and review of the literature

**DOI:** 10.1186/s12883-022-02618-0

**Published:** 2022-03-24

**Authors:** Alessandra Consales, Giulia Ardemani, Claudia Maria Cinnante, Mariana Rita Catalano, Claudia Giavoli, Roberta Villa, Maria Iascone, Camilla Fontana, Maria Francesca Bedeschi, Monica Fumagalli

**Affiliations:** 1 Fondazione IRCCS Ca’ Granda Ospedale Maggiore Policlinico, NICU, Milan, Italy; 2 University of Milan, Department of Clinical Sciences and Community Health, Milan, Italy; 3 Neuroradiology Unit, Fondazione IRCCS Ca’ Granda Ospedale Maggiore Policlinico, Milan, Italy; 4 Fetal Medicine and Surgery Service, Fondazione IRCCS Ca’ Granda, Ospedale Maggiore Policlinico, Milan, Italy; 5 Endocrinology Unit, Fondazione Istituto di Ricerca e Cura a Carattere Scientifico (IRCCS) Ca’ Granda Ospedale Maggiore Policlinico, Milan, Italy; 6 Clinical Genetics Unit, Fondazione IRCCS Ca’ Granda, Ospedale Maggiore Policlinico, Milan, Italy; 7 Laboratory of Medical Genetics, Ospedale Papa Giovanni XXIII, Bergamo, Italy

**Keywords:** Hypothalamic hamartoma, Pallister-Hall syndrome, cerebral ultrasound, brain MRI

## Abstract

**Background:**

Pallister-Hall syndrome (OMIM #146510) is a rare autosomal dominant condition caused by a mutation in the GLI3 gene. The cardinal feature of Pallister-Hall syndrome is the presence of hypothalamic hamartomas, which may manifest with seizures, panhypopituitarism and visual impairment. In Pallister-Hall syndrome, dysplastic histogenetic processes responsible for hypothalamic hamartomas are thought to disrupt early craniofacial development. The clinical presentation of Pallister-Hall syndrome may include: characteristic *facies* (low-set and posteriorly angulated ears, short nose with flat nasal bridge), cleft palate and uvula, bifid epiglottis and laryngotracheal cleft, limb anomalies (e.g., polysyndactyly, short limbs and nail dysplasia), anal atresia, genitourinary abnormalities and congenital heart defects.

**Case presentation:**

We report the case of two monochorionic diamniotic twins diagnosed with Pallister-Hall syndrome during the neonatal period, after the identification of a hypothalamic hamartoma on day 1 by cerebral ultrasound scan, later confirmed by brain magnetic resonance imaging. Cerebral ultrasound and magnetic resonance imaging presentations were identical in both twins.

**Discussion and conclusions:**

We review previously published cases (four reports) of hypothalamic hamartomas identified via cerebral ultrasound and compare reported ultrasonographic features. Main differential diagnoses based on cerebral ultrasound findings are discussed. Full description of typical magnetic resonance imaging appearance is also provided. This is the first case reported in the literature of monochorionic diamniotic twins affected by genetically confirmed Pallister-Hall syndrome with identical hypothalamic hamartomas at cerebral ultrasound and magnetic resonance imaging. Moreover, this paper adds to the existing literature on the sonographic appearance of hypothalamic hamartomas. Considering the consistency in hypothalamic hamartomas’ sonographic appearance, we support the use of cerebral ultrasound as a first-line neuroimaging modality in case of clinical suspicion of Pallister-Hall syndrome.

**Graphical Abstract:**

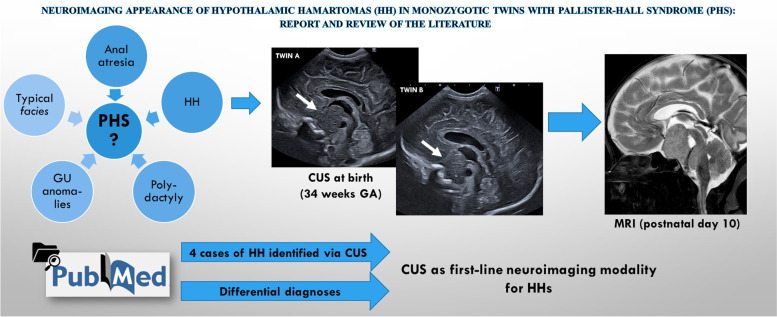

**Supplementary Information:**

The online version contains supplementary material available at 10.1186/s12883-022-02618-0.

## Background

Pallister-Hall syndrome (OMIM #146510, PHS) is a rare autosomal dominant condition (prevalence unknown) caused by a heterozygous mutation in the GLI3 gene, a zinc finger transcription factor gene located on chromosome 7p14.1 [1]. PHS was first described in 1980 as congenital hypothalamic hamartoblastoma syndrome [2]. Indeed, the cardinal feature of PHS is the presence of hypothalamic hamartomas (HHs). HHs are usually small (diameter 0.5–2 cm), slowly-growing malformations of grey matter composed of hyperplastic neurons located at the base of the brain in the third ventricular floor, near the tuber cinereum and the mammillary bodies. HHs of ≥40 mm in any dimension are considered “giant HHs” [3]. Due to their location in such an eloquent area of the brain, HHs may manifest with seizures, panhypopituitarism and visual impairment, as they progressively grow [4]. Based on neuroradiological imaging classification, HHs can be defined as sessile (or intrahypothalamic) or pedunculated (or parahypothalamic) [5]. The former ones surround and displace the hypothalamus and the third ventricle wall, whereas the latter ones are connected to the third ventricle floor or suspended from the inferior hypothalamus by a peduncle. Sessile HHs have been associated with gelastic epilepsy (frequently intractable), whereas pedunculated HHs are typically asymptomatic or present with signs of precocious puberty [4]. HHs in patients with PHS are not biologically aggressive and usually do not require neurosurgical treatment [6]. In PHS, dysplastic histogenetic processes responsible for HHs are thought to disrupt early craniofacial development leading to bilateral abnormalities of the midline. These include: low-set and posteriorly angulated ears, short nose with flat nasal bridge, cleft palate and uvula, bifid epiglottis and laryngotracheal cleft. Other features commonly described in patients with PHS are limb anomalies, such as polysyndactyly, short limbs and nail dysplasia. Imperforate anus and anal stenosis may also be found in patients with PHS. Genitourinary abnormalities have been reported as well, ranging from microphallus and cryptorchidism to renal hypoplasia or agenesis, and renal ectopia. Finally, patients with PHS may present congenital heart defects like patent ductus arteriosus, ventricular septal defect and proximal aortic coarctation [1].

The diagnosis of PHS is primarily clinical. The co-presence of a HH and meso-axial polydactyly is considered diagnostic. Other clinical features may support the diagnosis. For instance, bifid epiglottis is highly suggestive, given its rarity both in syndromes other than PHS and as an isolated malformation. Identification of a heterozygous pathogenic variant in GLI3 by molecular genetic analysis confirms the clinical diagnosis [1].

We present the case of two identical twins diagnosed with PHS during the neonatal period, after the identification of a HH by cerebral ultrasound (CUS) scan, later confirmed by brain magnetic resonance imaging (MRI). To the best of our knowledge, up to this day, only 4 cases of HHs identified via CUS have been described in the literature. We report two additional cases and compare their features with those previously reported. Furthermore, we summarize the main differential diagnoses to be considered in similar cases, and compare CUS findings.

## Case presentation

A 30-year-old primigravida with monochorionic diamniotic (MCDA) twin pregnancy was referred to our Fetal Medicine Unit at 12 weeks and 6 days for a second opinion due to a low-risk for chromosomal abnormalities first trimester screening scan showing a tubular anechoic area posterior to the bladder in one of the twins. Our gynecologists’ scan detected normal anatomy for gestational age in both twins. The following ultrasound (US) scans showed normal anatomy as well as amniotic fluid volume and Doppler studies in both twins, while fetal growth gradually dropped to the 17th and 7th centiles, respectively.

The twins were born by urgent caesarean section due to a preterm labor at 34 weeks' gestation, weighing 1900 and 1780 g, respectively.

**Fig. 1:**
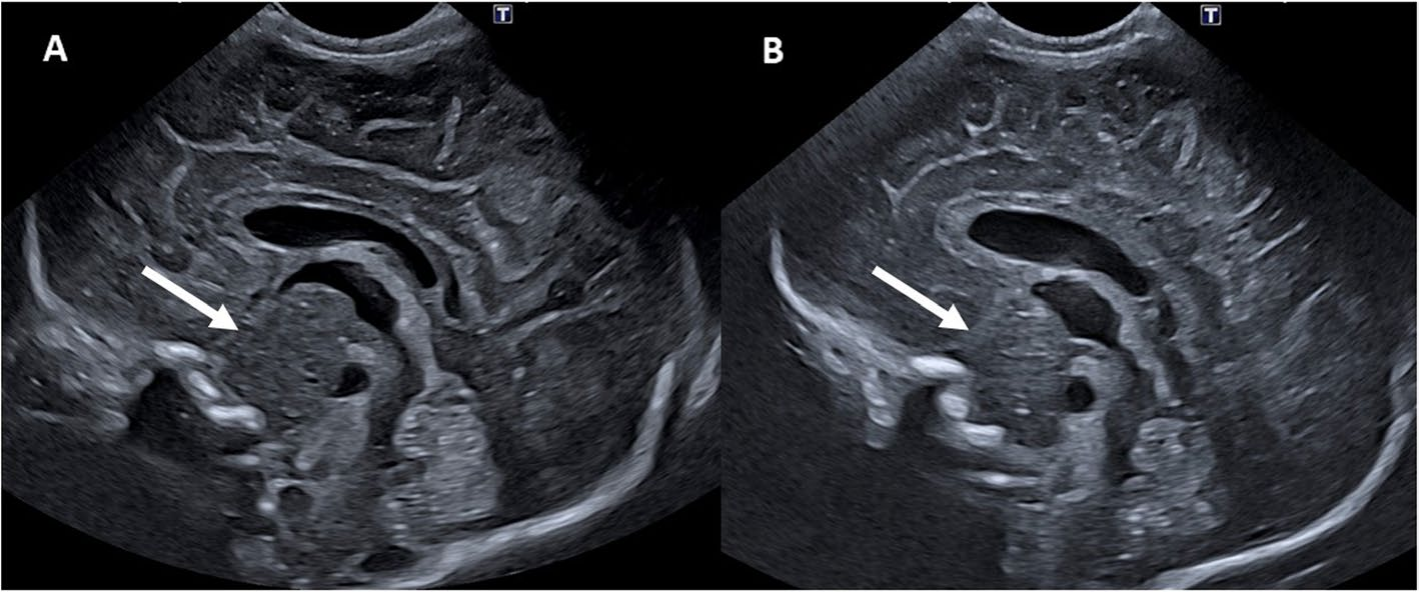
Mid-sagittal ultrasound scan through the anterior fontanel: **A** Twin A; **B** Twin B. White arrows indicate a round mass-like lesion in the suprasellar region, anterior to the brainstem, isoechoic to the surrounding parenchyma. The third ventricle’s floor is superiorly displaced, with a patent Sylvian aqueduct

**Fig. 2:**
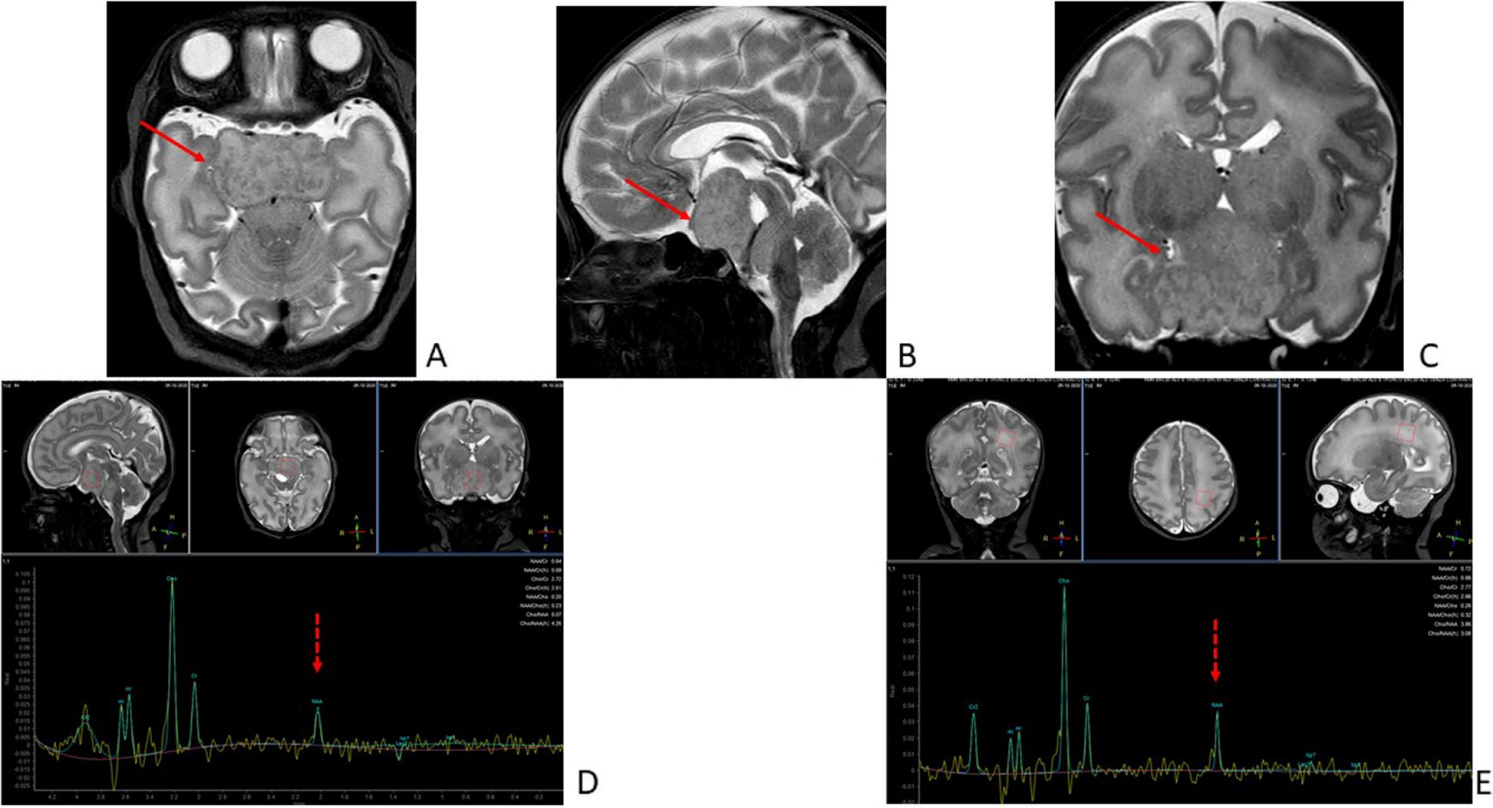
Brain MRI of Twin A performed at 35^+ 6^ weeks' gestation: TSE T2 axial (**A**), sagittal (**B**) and coronal (**C**) images showing a mass in the hypothalamic-tuber cinereum region (red arrow), with craniocaudal development from the third ventricle to the peripontine cisterns, characterized by a signal similar to the brain parenchyma. Brain MRS performed by PRESS technique with TE = 144 ms, with a single voxel placed in the center of the lesion (**D**), showing a slight reduction of N-acetylaspartate (NAA) within the lesion (dotted red arrow), compared to normal parenchyma (**E**), with single voxel placed in the periventricular posterior white matter (dotted red arrow)

Twin A was the first-born twin. Owing to mild respiratory distress at birth, the newborn was assisted with non-invasive ventilatory support, with progressive improvement of respiratory function. Anal atresia was discovered at birth and the infant was subsequently admitted to our Center’s neonatal intensive care unit (NICU). The initial physical examination also revealed peculiar *facies* (prominent forehead, sparse eyebrows, hypertelorism, depressed nasal root) (Suppl. Fig. 1), bilateral postaxial polydactyly of the hands with right IV-V digit syndactyly (Suppl. Fig. 2), and micropenis, without any additional genital abnormalities. Due to the prematurity and the presence of minor facial anomalies, on day 1 CUS was performed by an experienced neonatologist using an Aplio i700 Canon scanner (convex probe PVT-712BT, Frequency Range 4.3–11 MHz) (Fig. 1A). A mid-sagittal scan through the anterior fontanel demonstrated a round mass-like lesion (21.2 × 10.8 mm) in the suprasellar region, anterior to the brainstem, isoechoic to the surrounding parenchyma. The third ventricle floor was superiorly displaced, with a patent Sylvian aqueduct. Use of color Doppler imaging showed vascularization around - but not within - the lesion. A HH was suspected. Aside from axial hypotonus and overall hyporeactivity, Twin A’s neurological examination was unremarkable. Electroencephalography (EEG) showed a relatively well-organized activity pattern, and the newborn did not present seizures. Other relevant clinical features are described in Table 1. On post-natal day 10, a brain MRI was performed (Fig. 2) using a 3 T scanner (Achieva, Philips Healthcare, Best, The Netherlands) with pediatric-dedicated coil (Sense Ped, Philips Healthcare, Best, The Netherlands). The newborn was scanned during spontaneous sleep and monitored by pulse oximetry and electrocardiography. The MRI confirmed the presence of an expansive lesion (32x28x16 mm) in the hypothalamic-tuber cinereum region, with craniocaudal development from the third ventricle to the peripontine cisterns. The lesion was isointense with cerebral parenchyma. Magnetic resonance spectroscopy (MRS) showed a mild reduction of N-acetylaspartate (NAA) within the lesion, compared to normal parenchyma. The basilar artery and its branches appeared to be included in the lesion as well as both carotid siphons. The basilar artery had normal flow void signal, as if by progressive adaptation to the lesion. All things considered, the lesion was deemed compatible with a diagnosis of HH. The presence of a HH in a newborn with dysmorphic features supported a clinical diagnosis of PHS.

**Table 1:** Comparative clinical manifestations of Twin A and Twin B

Neonatal features	Twin A	Twin B
**Anthropometric measures (percentile)**	BW 1900 g (19th), L 43 cm (17th), CC 29 cm (3rd)	BW 1780 g (12th), L 41 cm (4th), CC 31 cm (29th)
**Facies**	Prominent forehead, sparse eyebrows, hypertelorism with divergent strabismus, depressed nasal root	Prominent forehead, sparse eyebrows, hypertelorism with divergent strabismus, depressed nasal root
**Limbs**	Bilateral post-axial polydactyly type B, IV-V finger syndactyly of the right hand, left clubfoot	Right hand post-axial polydactyly type B
**Heart**	Two left ventricular false tendons	Left ventricular false tendon
**Genito-urinary system**	CAKUT with chronic renal failure, 2nd grade bilateral VUR; micropenis	Transient bilateral calico-pyelic dilatation and of the proximal ureter
**GI tract**	Anal atresia	Anal atresia
**ENT**	Omega-shaped epiglottis, mild laryngomalacia	Normal
**Endocrinological findings**	GHD	Subclinical hypothyroidism (with normal thyroid gland US), GHD
**Seizures**	No	No
**EEG**	Normal	Anomalies in the temporal region (with asymmetry, left>right): slow waves both isolated and in sequences, sometimes in the form of sharp waves.
**Ophthalmological assessment**	Normal	Normal
**Audiological screening**	Normal	Normal
**CUS**	Fig 1A	Fig 1B
**Spinal US**	Cyst of the filum terminale	Normal
**MRI**	Fig 2	Fig 3A/B

*Abbreviations*: *BW* Birthweight, *L* Length, *CC* Cranial circumference, *CAKUT* Congenital anomalies of the kidney and urinary tract, *VUR* Vesicoureteral reflux, *GI* Gastro-intestinal, *ENT* Ear-nose-throat, *GHD* Growth hormone deficiency, *US* Ultrasound, *CUS* Cranial ultrasound, *MRI* Magnetic resonance imaging, *EEG* Electoencephalography

**Fig. 3:**
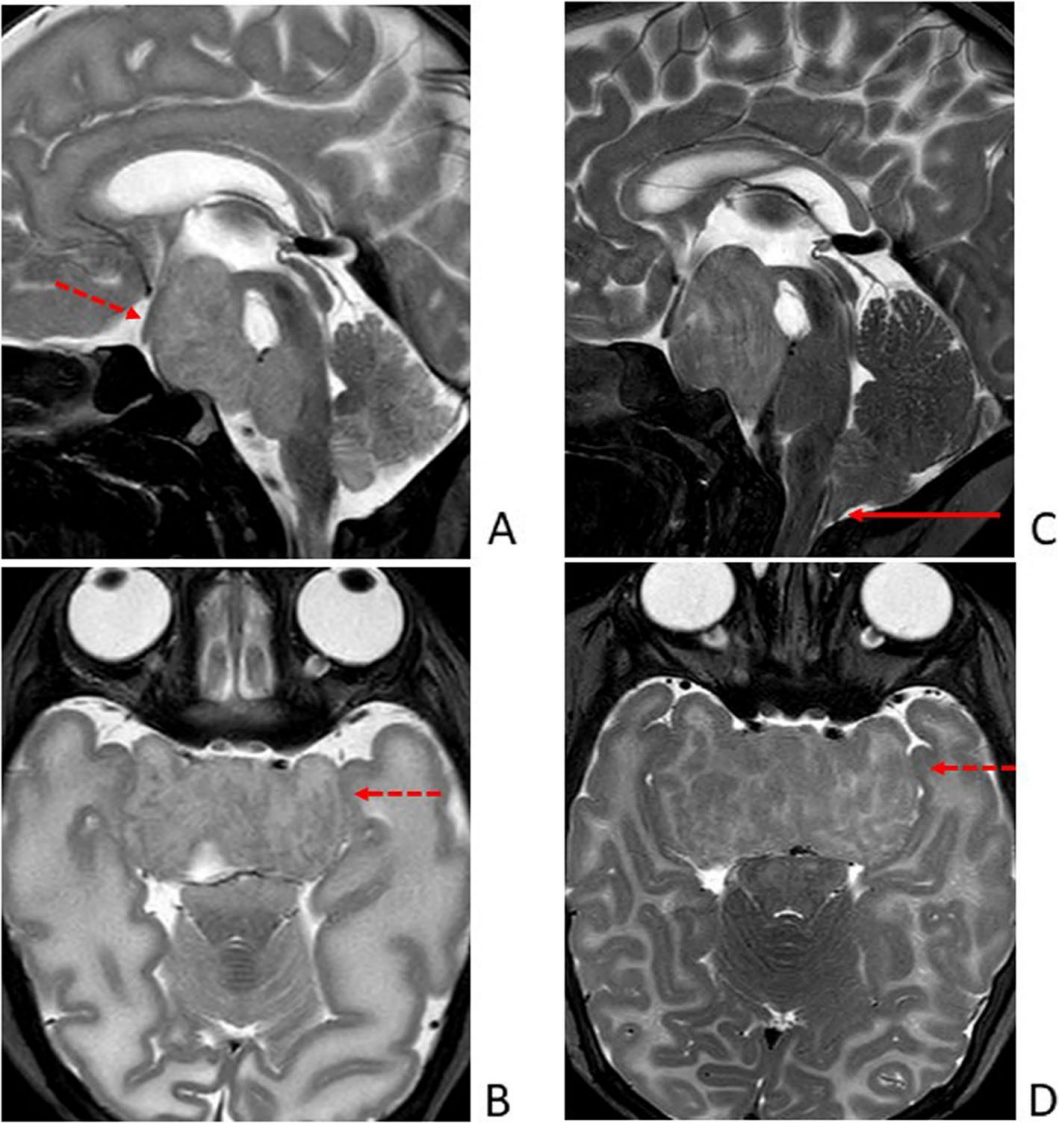
Brain MRI of Twin B comparing TSE T2 sagittal (**A**) and axial (**B**) images performed at 35^+ 6^ weeks' gestation, with the same images (**C**, **D**) performed at 50^+ 6^ weeks of corrected age: the lesion (dotted red arrow) increased in size causing a more relevant mass effect on the surrounding structures and an initial displacement of the cerebellar tonsils through the foramen magnum (red arrow). The lesion still appeared isointense to the surrounding parenchyma, with myelination-like aspects and thickening of the intra-lesional cortical component

Twin B was the second-born twin. He developed mild respiratory distress at birth requiring non-invasive ventilatory support for the first hours after birth. Similarly to his brother, anal atresia was discovered at birth and he was admitted to our NICU. Peculiar minor facial anomalies similar to Twin A’s were noted. However, Twin B presented polydactyly only of the right hand. Comparison between the main clinical features of the twins is summarized in Table 1. The main differences were observed from a nephrological and endocrinological point of view. Interestingly, CUS and MRI findings were superimposable (Figs. 1B and 3A/B).

The clinical diagnosis of PHS was confirmed in both twins by whole exome sequencing (WES), which showed the same variant p.Thr694fs in heterozygosity (NM_000168:c.2080del), caused by a deletion of a base in exon 13 of the GLI3 gene. Sanger sequencing of peripheral blood DNA from the parents did not detect the GLI3 variant.

The twins underwent sequential CUS scans and at 50^+ 6^ weeks of corrected age brain MRI was performed to assess the HHs’ size and potential associated complications. In both twins, the known expansive lesion appeared enlarged (approximately 50% on the antero-posterior and latero-lateral diameter), causing a more relevant mass effect on the surrounding structures. In Twin B, an initial displacement of the cerebellar tonsils through the foramen magnum was observed (Fig. 3C/D). The lesions still appeared isointense to the surrounding parenchyma. Myelination-like aspects within the HHs and thickening of the intra-lesional cortical component could be recognized. Compared to the previous exam, on MRS NAA content appeared physiologically increased within the periventricular white matter. A slight increase in NAA, although less marked, was detected within the lesions, as well.

## Discussion and conclusions

As far as we know, this is the first reported case of MCDA twins affected by genetically confirmed PHS. Moreover, this paper adds to the existing literature on the sonographic appearance of HHs, describing two identical CUS presentations, confirmed by MRI.

In 1991, Hingorani et al. [7] described the case of MCDA twin female fetuses aborted at 145 days of gestational age, concordant for oral, facial, skeletal, and central nervous system malformations. The malformations observed were considered an overlap between the oral-facial-digital syndrome, hydrolethalus syndrome, and PHS. Interestingly, both fetuses presented a large bosselated tissue mass replacing the third ventricle, ventral thalamus and hypothalamus, protruding from the basal surfaces and compressing the anterior brainstem. The microscopic examination identified the masses as hamartomas. Amniocyte chromosomes of one of the two fetuses were normal; no other genetic analysis was performed.

In our case, Trio exome analysis identified the de novo heterozygous variant p.Thr694fs in the GLI3 gene. The variant has not been described in the literature. Most pathogenic variants that cause PHS are frameshift variants, as in the present case. For this reason, it is likely to be considered pathogenetic. The twins’ parents are in good general health and do not present the classic features of PHS (no specific *facies* nor polysyndactyly).

In the present case, CUS findings, later confirmed by MRI, represented an important diagnostic handle that strengthened our clinical suspicion of PHS and led subsequent examinations. Indeed, HHs are a specific feature of PHS and MRI is currently considered the modality of choice for their diagnosis. On MRI HHs have a characteristic appearance [8]: they are non-calcified and non-enhancing lesions, homogeneously isointense to gray matter on T1-weighted images and often hyperintense on T2-weighted images. On MRS, a reduction in NAA content within the lesion and a parallel increase in myoinositol appears, suggesting decreased neuronal density and relative gliosis compared with normal gray matter [9]. These imaging findings are helpful in differentiating HHs from other more common suprasellar lesions such as craniopharyngiomas and hypothalamic/opticochiasmatic gliomas [9, 10]. Twin A and Twin B’s MRI findings were consistent with such appearance.

Given its distinctive features, a prenatal diagnosis of HH by fetal MRI is also feasible, as recently described by Cristobal et al. [11], who highlighted the added value of multimetric analysis using different sequences. In fact, thanks to its higher-contrast resolution compared with prenatal sonography, fetal MRI allows for a better visualization of fetal brain development and detection of intracranial abnormalities [12]. However, fetal MRI is not routinely performed but only requested in case of prenatal US suspicion of brain anomalies. In our case, prenatal US examinations failed to detect intracranial abnormalities, most probably because of the location of the lesions and their isoechoic appearance, that further challenges US diagnosis as they are hardly distinguishable from the normal cerebral parenchyma. Conversely, postnatal CUS detection of HHs was driven by the clinical suspicion of PHS based on the congenital anomalies observed at birth. Furthermore, the complexities of US imaging in a twin pregnancy may at least partly account for the missed prenatal identification of the HHs, together with the observation that HHs are slowly-growing malformations that probably became more obvious with increasing gestational age. Hence, in our case a fetal MRI was not performed.

MRI is also the modality of choice for long-term follow-up. The absence of changes over time in a suspected HH is considered a defining feature [13]. In our case, in both twins the lesions appeared to be increased in size and “more mature” at 2½ months corrected age. One possible explanation for this finding is the fact that in our twins HHs were diagnosed at 35^+ 6^ weeks' gestation, after a premature birth. HHs are composed by grey matter and hyperplastic neurons and, similarly to cerebral tissue, are expected to increase in size during the first months of life alongside the surrounding brain. Indeed, the last half of human gestation is characterized by active brain growth [14]. In addition, an overall cerebral growth of 64% in the first 90 days after term birth has been described [15], with the cerebellum being the fastest growing structure (around 100% in 3 months) [15–17]. HHs’ maturational nature has been previously demonstrated through histologic examinations. Indeed, the initial denomination “hamartoblastoma” was due to the reported presence of primitive undifferentiated germinal cells in the first lesions biopsied. However, as longer survival was achieved through prompt recognition and improved intensive and supportive care, a less primitive appearance could be appreciated [18].

As opposed to MRI, CUS documentation of HH is not common. This may be explained by the fact that HHs are more likely diagnosed when the first symptoms appear, specifically precocious puberty or gelastic seizures, usually later in childhood, when CUS can no longer be performed due to the closure of the cranial fontanels. Table 2 summarizes the 4 cases of HHs identified via CUS previously described in the literature and compares them to our findings.

**Table 2:** Review of all reported cases of hypothalamic hamartomas visualized through cranial ultrasound

Author (year)	GA at birth (weeks)	Age at diagnosis	Neuroimaging									Histological confirmation	PHS Diagnosis
			Prenatal Examination	Postnatal Examination									
				CUS					MRI				
				Location	Size (cm)	Echogenicity	Hydrocephalus	Mass effect	Size (cm)	Signal	MRS		
Martijn (1984) [19]	n.a.	4 months	n.a.	n.a.	2	Hyperechoic	yes	yes	n.a.	n.a.	n.a.	n.a.	no
Guibaud et al. (1995) [20]	Term	14 days	n.a.	Midline, anterior to the posterior fossa	4.6 × 3.3	Hyperechoic	no	yes	5	T1: isontense, T2: eterogeneously hyperintense. Nonenhancing	n.a.	yes, examination of surgical specimen	no
Kos et al. (2008) [21]	36	n.a.	n.a.	Hypothalamus	2.5	Isoechoic	no	n.a.	n.a.	Isointense on all sequences. Nonenhancing.	n.a.	n.a.	yes, GLI3 mutation (Q717X)
Joo Yeon Lee et al. (2016) [22]	36	n.a.	28 weeks GA: arachnoid cyst	Anterior to the left temporal lobe	5.1 × 3.5	Isoechoic	no	yes	6 × 3 × 4.3	Isointense on all sequences. Nonenhancing. Few areas with slightly high T1 signal intensity.	Chemical composition similar to white matter	yes, surgical biopsy	no
Present Case	34	10 days	Unremarkable	Suprasellar region, anterior to the brainstem	2.1 × 1	Isoechoic	no	Displacement of third ventricle floor	3.2 × 2.8 × 1.6	Isointense	Mild reduction of NAA	no	yes, GLI3 heterozygous mutation (p.Thr694fs)

*Abbreviations*: *GA* Gestational age, *CUS* Cranial ultrasound, *MRI* Magnetic resonance imaging, *MRS* Magnetic resonance spectroscopy, *PHS* Pallister-Hall syndrome; n.a., data not available; NAA, N-acetylaspartate

Despite having been almost unanimously described as well-defined homogenous lesions in a typical location, determining mass effect but usually not hydrocephalus, differential diagnosis with other suprasellar lesions must be considered.

Brain tumors in the neonatal period are uncommon compared to older children and adults. Their sonographic appearance may vary and complex echogenic patterns are frequently found; hydrocephalus is commonly present [23]. Among suprasellar tumors, teratomas usually appear at CUS as well-defined, round, midline masses occupying the cerebral hemispheres, less frequently within the pineal gland or the third ventricle. Due to the presence of calcifications, fat inclusions and soft tissue within the lesion, they typically present mixed echogenicity. Cystic components are common and probably represent necrotic areas in rapidly-growing tumors [24]. In the case of hypothalamic pilocytic astrocytomas, CUS usually shows lobulated and bulky masses, homogeneously hyperechoic and frequently causing displacement of the third ventricle and midbrain structures [25]. Craniopharyngiomas rarely present in the neonatal period, therefore their CUS characteristics have not been frequently described. In 1988, Hurst et al. [26] described a craniopharyngioma in a 1-day-old newborn, presenting at CUS as a suprasellar heterogeneous mass with hyperechoic shadowing images compatible with calcifications; hydrocephalus was also reported. Likewise, intracranial lipomas are rare and have seldom been described in neonates but their CUS appearance is typically that of a hyperechoic mass [27], often associated with anomalies of the corpus callosum. The echogenic pattern and general characteristics of the aforementioned tumors differ from what was documented in Twin A and Twin B. Nonetheless, a brain MRI is required to confirm a diagnosis of HH and exclude possible differential diagnoses.

Color and spectral Doppler imaging may aid in the differential diagnosis of cerebral lesions in the newborn. In particular, by mapping blood flow velocity within a region of interest, they allow the evaluation of suspected vascular lesions [28, 29]. In our case, Color Doppler image showed flow around - but not within - the lesion, thus excluding a vascular anomaly.

The present case highlights the importance of a multidisciplinary team approach in the management of complex newborns. Indeed, it was mainly due to the well-oiled teamwork between neonatologists, neuroradiologists and clinical geneticists, each with their own expertise, that a diagnosis was promptly reached.

The early execution of an admission CUS, within this specific clinical context, allowed the timely identification of suspected HHs, which led the subsequent diagnostic process. CUS is the first-line neuroimaging modality to study the neonatal brain and a clinically-driven early CUS is paramount for further directing diagnostics. Compared to MRI, CUS can be performed at the patient’s bedside, immediately after birth and provides real-time images that can be used to monitor brain development and lesions over time. In the last decade, the quality of CUS has dramatically improved, allowing the appropriately trained neonatologist to promptly recognize a variety of brain lesions both in the term and preterm infant [30], although its helpfulness is still limited by the skills, knowledge and experience of the operator. Therefore, CUS and MRI are complementary techniques, although MRI remains the gold-standard for diagnosing neonatal brain abnormalities, particularly in case of brain malformations, by providing a detailed description of anatomical features.

In conclusion, this is the first case reported in the literature of MCDA twins with genetically confirmed PHS, whose diagnosis was suggested by the identification of findings consistent with HHs by CUS. CUS findings were identical in both twins and were later confirmed by brain MRI. Thanks to the multidisciplinary approach, the identification of a suprasellar mass consistent with a diagnosis of HH, in the presence of suggestive clinical findings, raised the suspicion of PHS and guided subsequent evaluations. Furthermore, we demonstrated how the twins’ CUS imaging appeared in line with previously described HHs. Given the consistency in HHs’ sonographic appearance, we support the use of CUS as a first-line neuroimaging modality for suspected HHs.

## Supplementary Information

**Additional file 1: Suppl. Fig. 1.** Facial appearance of Twin A (**A**) and Twin B (**B**) at birth showing typical features of PHS: frontal bossing macrocephaly, hypertelorism, broad flat nasal bridge, anteverted nares and small upper lip and philtrum. Both twins had anal atresia.

**Additional file 2: Suppl. Fig.** **2.** Hand view of Twin A showing typical IV-V digit syndactyly and postaxial type A polydactyly.

## Data Availability

Not applicable.

## Abbreviations

CUS: Cerebral ultrasound
EEG: Electroencephalography
ENT: Ear-Nose-Throat
GHD: Growth hormone deficiency
MCDA: Monochorionic Diamniotic
MRI: Magnetic Resonance Imaging
MRS: Magnetic Resonance Spectroscopy
NAA: N-acetylaspartate
NICU: Neonatal Intensive Care Unit
HH: Hypothalamic hamartoma
PHS: Pallister-Hall syndrome
US: Ultrasound
VUR: Vesicoureteral reflux
WES: Whole Exome Sequencing
