# Supplementary figures and images for: Neuroimaging appearance of hypothalamic hamartomas in monozygotic twins with Pallister-Hall syndrome: case report and review of the literature
Source: BMC Neurol. 2022 Mar 24;22:118. doi: 10.1186/s12883-022-02618-0 (PMC8943937; doi:10.1186/s12883-022-02618-0)

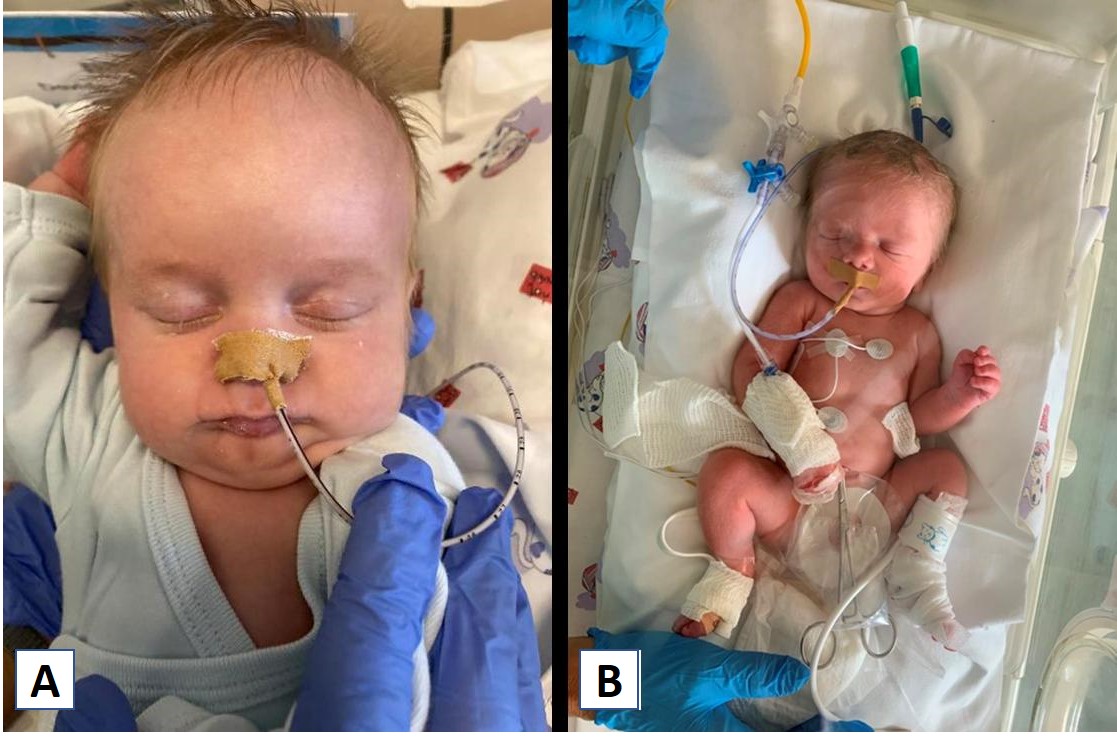

Supplement: Supplementary file 1 — Additional file 1: Suppl. Fig. 1. Facial appearance of Twin A (A) and Twin B (B) at birth showing typical features of PHS: frontal bossing macrocephaly, hypertelorism, broad flat nasal bridge, anteverted nares and small upper lip and philtrum. Both twins had anal atresia. [file 12883_2022_2618_MOESM1_ESM.jpg]

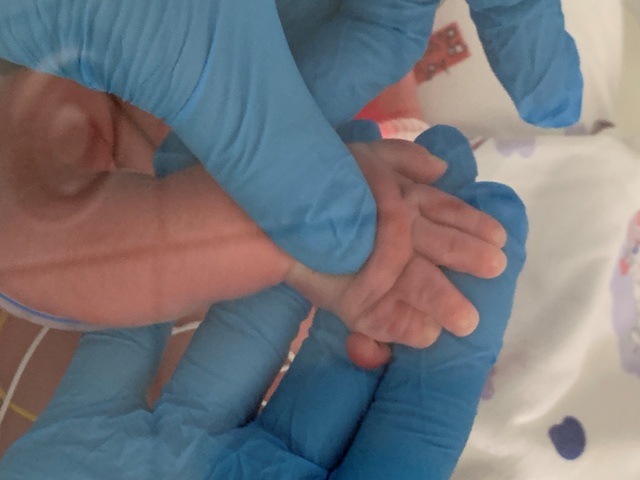

Supplement: Supplementary file 2 — Additional file 2: Suppl. Fig. 2. Hand view of Twin A showing typical IV-V digit syndactyly and postaxial type A polydactyly. [file 12883_2022_2618_MOESM2_ESM.jpg]
